# Hall and Ion-Slip Effect on CNTS Nanofluid over a Porous Extending Surface through Heat Generation and Absorption

**DOI:** 10.3390/e21080801

**Published:** 2019-08-16

**Authors:** Ibni Ameen, Zahir Shah, Saeed Islam, Saleem Nasir, Waris Khan, Poom Kumam, Phatiphat Thounthong

**Affiliations:** 1 Department of Mathematics, Abdul Wali Khan University, Mardan 23200, Pakistan; 2 Center of Excellence in Theoretical and Computational Science (TaCS-CoE), SCL 802 Fixed Point Laboratory, Science Laboratory Building, King Mongkut’s University of Technology Thonburi (KMUTT), Bangkok 10140, Thailand; 3 KMUTT Fixed Point Research Laboratory, Room SCL 802 Fixed Point Laboratory, Science Laboratory Building, Department of Mathematics, Faculty of Science, King Mongkut’s University of Technology Thonburi (KMUTT), Bangkok 10140, Thailand; 4 Department of Mathematics, Kohat University of Science & Technology, Kohat 26000, Pakistan; 5 KMUTT-Fixed Point Theory and Applications Research Group, Theoretical and Computational Science Center (TaCS), Science Laboratory Building, Faculty of Science, King Mongkut’s University of Technology Thonburi (KMUTT), Bangkok 10140, Thailand; 6 Department of Medical Research, China Medical University Hospital, China Medical University, Taichung 40402, Taiwan; 7 Renewable Energy Research Centre, Department of Teacher Training in Electrical Engineering, Faculty of Technical Education, King Mongkut’s University of Technology North Bangkok, 1518 Pracharat 1 Road, Bangsue, Bangkok 10800, Thailand

**Keywords:** porous medium, SWCNTs/MWCNTs, kerosene oil, stretching sheet, rotating system, hall and ion-slip, HAM

## Abstract

In this research work, a 3D rotating flow of carbon nanotubes (CNTs) over a porous stretchable sheet for heat and mass transfer is investigated. Kerosene oil is considered as a base liquid and two types of CNTs, (Single & Multi) WCNTs are added as the additives to the base liquid. The present analysis further comprises the combined effect of the Hall, ion-slip, and thermal radiation, along with heat generation/absorption. The appropriate ordinary differential system of equations after applying appropriate transformation is calculated. The resulting nonlinear system of equations (conservation of mass, momentum, temperature) is explained by HAM (Homotopy Analysis Method). Solution of velocities and thermal fields are obtained and discussed graphically. Expression of $C_{f}$ and Nu are intended for both type of nanoliquids. The influences of prominent physical factors are plotted for velocities and thermal profiles using Methematica. These graphical results are qualitatively in excellent agreement with the previous published results. Also, single wall nanoparticles are found to have higher temperatures than multi wall CNTs nanoparticles.

## 1. Introduction

Practically heat transport phenomena plays a significant role in several chemical, biological, and mechanical processes. Heat generated through any manufacturing process needs to be ejected uniformly from the setup to increase the capability of a manufacturing setup for a long interval. Weak thermal conductivity of ordinary liquids such as water and various oils can be a big difficulty for attaining growing thermal conductivity of any mechanical mechanism. As a result, there are numerous techniques present in the literature to alleviate the problems caused by the heat exchange and thermal efficiency of common liquids. There are various real-life functioning liquids such as oils, H_2_O (water), and C_2_H_6_O_2_ (ethylene glycol) that have comparatively weak thermal conductivities in contrast to solids. To improve the thermal efficiency of these common liquids, fine and tiny type solids materials are used. These tiny form materials are called nanoparticles. Consequently, the homogeneous mixture of nano-meter (1 nm–100 nm) size particles in functioning liquids is called a nanofluid. The pioneered concept of nanoliquid was suggested by Choi in 1995 [1]. Nanofluid, characterized by a significant increase in thermal conductivity compared to conventional engineered fluid [1], is found to serve in a number of engineering applications, such as porous materials [2], the fuel-cell industry [3], petroleum engineering [4], etc. Xu and Pop [5] studied the properties of gyrotactic micro-organisms in the stagnation point flow of viscid nanofluid on an extending plate. Das et al. [6] described the flow of viscid nanofluids with thermal radiation on a stretching medium. Nadeem et al. [7] scrutinized the water base nanofluids flowing through an extending medium numerically. Gireesha and Ramesh [8] inspected the flow of Maxwell nanoliquid along with heat generation (source and sink). Ellahi et al. [9] considered the features of entropy generation, including nonlinear radiation, using MHD Cu-H_2_O nanofluid. Sheikholeslami and Ellahi [10] scrutinized hydrothermal behavior in MHD flow nanofluid. In recent times, some notable analysis in this platform can be seen in References [11,12,13]. After having examined nanofluids in the aforementioned review, it is worth noting here that the effect of Hall and ion-slip non-linear thermal radiation, along with heat generation/absorption in three-dimension rotating flow of nanofluids, is implemented in present analysis, which has never been done before.

In this analysis, the author determined that carbon nanotubes exhibit remarkable mechanical and thermal features. This is due to such exceptional features that make CNTs useful for nanotechnology and nanoscience with biochemical manufacture, optics, and specialized fields in manufacturing and physical sciences. Actually, carbon nanotube (CNTs) is the allotrope of carbon in a tube (pipe), which is designed to have substantial effects with a cylinder-shaped nano structure. The pioneering idea of CNTs was first provided by Ijima and Baughman [14] in 1991. Haq et al. [15] scrutinized the convective heat transfer with MHD slip flow in presence of CNTs nanoliquids. In this analysis of CNTs, nanoparticles perform a dynamic role in the enhancement of the thermal efficiency of based liquid. Mahanthesh et al. [16] examined the MHD flow of CNTs-water nanoparticles in the presence of Marangoni convection and thermal radiation due to the stretching disk. They used the RK method through a shooting scheme to obtain the numerical results of nonlinear expression. Hayat et al. [17] presented the impact of heat flux on a chemically reactive unsteady flow of CNTs nanoliquids containing both SWCNTs and MWCNTs nanoparticles. Their results show that the Nusselt number increase due to an increasing magnitude of curvature and thermal relaxation factors. The influence of convective physical condition on the flow of SWCNTs and MWCNTs nanofluids over a vertical cone is examined by Sreedevi et al. [18]. Jyoti et al. [19] considered the CNTs (SWCNTs and MWCNTs) nanofluids with MHD and convective boundary conditions between two rotating and stretchable disks. More recently Nasir et al. [20,21,22] discussed the 3D rotating flow of SWCNTs over a stretching sheet and disk. Furthermore, some current related investigation in this regard can be perceived in the analysis [23,24].

Generally, in the study of MHD flows, the Hall current and also ion slip relations in Ohms law have been neglected in order to easily conduct mathematical investigation of the flow. But, the consequence of the Hall current and ion slip is very important in the presence of a high magnetic field. So, in numerous natural circumstances, it is essential to comprise the effect of Hall current and ion slip terms of the magneto-hydrodynamics expressions. In 1962, Tani [25] studied the effects of Hall term on the steady flow of electrically conducting nanofluid. Hayat et al. [26] presented the effects of ion slip, Hall current along with nonlinear radiation on the flow of nanofluids in a channel. Srinivasacharya and Shafeeurrahmann [27] analyzed the impact of ion-slip and Hall parameters on the flow of nanofluids between two tubes. Moreover, some current related study in this regard can be supposed in the investigation [28,29,30,31]. Ellahi et al. [32,33] shaped the factor of nanofluid. Some related work can be seen in references [34,35,36,37,38]. Yikun Wei et al. [39,40,41,42,43,44,45,46] investigated thermal behavior, heat transfer, and entropy using the Lattice Boltzmann Method and also a numerical approach.

All of the analysis of the past has considered different characteristics and properties of fluids over a stretching sheet. Little study has been done on carbon nanotubes based nanofluid and heat transport through porous stretching sheet with Hall current and an ion-slip effect. We fill such gaps and present some interesting results on these topics. Therefore, the central aim of the present investigation is to find the influence of Hall current and the ion-slip effect on the three-dimensional rotating flow of CNTs nanoliquid through a stretching porous sheet. Heat transport analysis is explored using heat generation/absorption and non-linear thermal radiation. Two types of CNTs nanoparticles (SWCNTs, MWCNTs) are utilized in kerosene oil. The resulting nonlinear expressions are solved by using the Homotopy analysis technique (HAM) technique [47,48,49]. HAM is an important and highly prevalent method used to solve these types of problems. It is a semi-analytical technique used to solve nonlinear ordinary/partial differential equations. Many researchers [50,51,52,53,54,55,56,57,58] have used it due to its fast convergence.

The current article has been organized as flows. In Section 2, the formulation of flow and heat problems are presented. The solution for the problem has been discussed in Section 3. In Section 4, the influences of some appropriate factors are deliberated through graphs and tables. Also, the physical appearance of skin friction and Nusselt numbers corresponding to different parameters are examined through numerical and graphical data. The conclusion has been drawn in Section 5.

## 2. Formulation of the Problem

**Figure 1:**
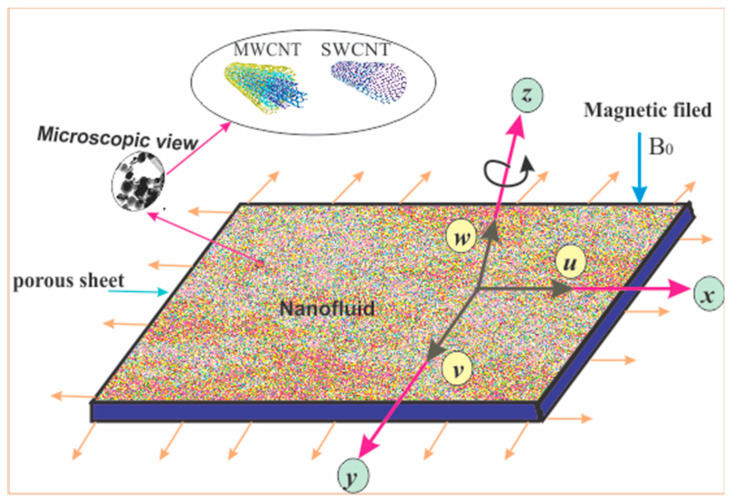
Physical model of the flow problem.

Consider an incompressible rotating flow of electrically conducting CNTs nanoliquid past a porous extending sheet. The flow of nanoliquid is in an steady state and is three dimensional. The nanoliquid in current exploration is composed of carbon nanotubes (CNTs), nanoparticles, SWCNTs, and MWCNTs added to a base liquid (kerosene oil) [20]. The graphical view of the problem, the physical explanation, and the coordinate system of such a flow model are shown in Figure 1. A strong continuous magnetic field $B_{0}$ has been assumed. In this scenario, the collision rate of the electron atom is supposed to be comparatively high; therefore, the impression of Hall current along with ion-slip effects cannot be ignored as in reference [29]. The s>0, is the stretching rate of the sheet along x-direction, wherever the rotation if the fluid is performed in the z-axis. The constant angular velocity of liquid is represented by ϖ. Furthermore, the nonlinear thermal radiation and heat generation/absorption are also considered. The surface temperature of plate is due to the convection heat mechanism that is included through hot liquid temperature $\left(T_{f}\right)$ and $\left(h_{f}\right)$ is the heat transfer coefficient. 

For analysis of Hall current, the generalized form of Oham’s law has been used [29,30,31,32,33,34,35,36,37,38] in the form

(1) $$J=\frac{1}{1+{\left(\frac{\omega }{v_{e}}\right)}^{2}}\left[\sigma \left(E+\left(V\times B\right)\right)-\frac{\sigma \left(J\times B\right)}{e\;\alpha_{e}}\right].$$

Here, the electron cyclotron is denoted by ω and the collision frequency of electron atom is denoted by $v_{e}$. The phenomena of ion-slip happens when the relation $\frac{\omega }{v_{e}}$ becomes very large.

(2) $$\frac{\partial \;u}{\partial \;x}+\frac{\partial \;v}{\partial \;y}+\frac{\partial \;w}{\partial \;z}\;=0,$$

By applying the above-mentioned assumption, the governing equations become [21,28]:

(3) $$\rho_{nf}\left[u\frac{\partial \;u}{\partial \;x}+v\frac{\partial \;u}{\partial \;y}+w\frac{\partial \;u}{\partial \;z}-2\;\left(v\right)\;\varpi \right]=\mu_{nf}\left[\frac{\partial^{2}u}{\partial \;z^{2}}\right]-\frac{\sigma_{nf}\;B_{0}^{2}\left(v\;m_{e}-u\;n_{e}\right)\;}{\rho_{nf}\left(m_{e}^{2}+n_{e}^{2}\right)}-\frac{1}{\rho_{nf}}\left(\frac{\mu_{nf}}{k^{⊗}}\right)u,$$

(4) $$\rho_{nf}\left[u\frac{\partial \;v}{\partial \;x}+v\frac{\partial \;v}{\partial \;y}+w\frac{\partial \;v}{\partial \;z}+2\left(u\right)\;\varpi \right]=\mu_{nf}\left[\frac{\partial^{2}v}{\partial \;z^{2}}\right]-\frac{\sigma_{nf}\;B_{0}^{2}\left(v\;m_{e}-u\;n_{e}\right)\;}{\rho_{nf}\left(m_{e}^{2}+n_{e}^{2}\right)}-\frac{1}{\rho_{nf}}\left(\frac{\mu_{nf}}{k^{⊗}}\right)v,$$

(5) $$\begin{array}{l} u\;\left(\frac{\partial T}{\partial x}\right)+v\;\left(\frac{\partial T}{\partial y}\right)+w\;\left(\frac{\partial T}{\partial z}\right)=\alpha_{nf}\;\left(\frac{\partial^{2}T}{\partial z^{2}}\right)+\frac{1}{{\left(\rho c_{p}\right)}_{nf}}\left[\frac{\sigma_{nf}\;B_{0}^{2}\;}{\left(m_{e}^{2}+n_{e}^{2}\right)}\left(u^{2}+v^{2}\right)-\left(\frac{\partial q_{rad}}{\partial z}\right)\right]\\ +\frac{Q(T-T_{0})}{{\left(\rho c_{p}\right)}_{nf}}+\frac{\mu }{{\left(\rho c_{p}\right)}_{nf}}{\left(\frac{\partial u}{\partial z}\right)}^{2}. \end{array}$$

Here, $m_{e}=1+n_{i}n_{e}$, where $n_{i}$ is denoted the ion-slip while $n_{e}$ is the Hall current parameters. The appropriate boundary conditions of the problem at z=0 and z=∞ are as follows [16,20]:

(6) $$\begin{array}{l} u=s\;x=u_{w},\;v=\;0=w,\;-\left[\frac{\partial T}{\partial z}\right]=\frac{h_{f}}{k_{nf}}\left(T_{f}-T\right)\;,\;at\;z=0,\\ u,\;v\to \;0,\;T\to \;T_{\infty }\;,\;at\;z=\infty . \end{array}$$

Here the components of velocities in x,y,z directions imply u,v,w, respectively. Similarly, $\rho_{n\;f},\;\mu_{n\;f},\;\sigma_{n\;f},\;T,\;k_{n\;f},\;{\left(\rho c_{p}\right)}_{n\;f}$ implies density, dynamic viscosity, electrical conductivity, local temperature, the thermal conductivity, and the specific heat capacity of nanoliquid, respectively. The aforementioned parameters are defined as [22,23]:

(7) $$\begin{array}{l} \rho_{nf}=\rho_{f}\left[1-\chi +\frac{\rho_{CNT}}{\rho_{f}}\chi \right],\;\mu_{nf}=\mu_{f}{\left(1-\chi \right)}^{-\frac{5}{2}},\;\alpha_{nf}=\frac{\mu_{f}}{{\left(\rho \;c_{p}\right)}_{nf}},\\ \;\frac{{\left(\rho \;C_{p}\right)}_{nf}}{{\left(\rho \;C_{p}\right)}_{f}}=\left[1-\chi +\frac{\chi \left(\rho \;C_{p}\right){}_{CNT}}{{\left(\rho \;C_{p}\right)}_{f}}\right]\;,\sigma_{nf}=\sigma_{f}\;\left(\frac{1+3\left(\sigma -1\right)\;\chi }{\left(\sigma +2\right)-\left(\sigma -1\right)\chi }\right). \end{array}$$

Here, the Xue [38] model has been applied to determine the thermal conductivities

(8) $$\frac{k_{nf}}{k_{f}}=1-\chi +2\chi \;\left(\frac{k_{\;CNT}}{\left(k{\;}_{CNT}-k_{f}\right)}\;ln\;\frac{k{\;}_{CNT}+k_{f}}{2\;k_{f}}\right){\left\{1-\chi +2\chi \;\left(\frac{k_{f}}{\left(k{\;}_{CNT}-k_{f}\right)}\;ln\;\frac{k{\;}_{CNT}+k_{f}}{2\;k_{f}}\right)\right\}}^{-1}.$$

where χ reveal the nanoparticle volume fraction. Also $\rho_{f},\;\mu_{f},\;\sigma_{f},\;k_{f},\;{\left(\rho c_{p}\right)}_{f}$ implies density, viscosity, electrical and thermal conductivities, and the specific heat capacity of base (common liquid), respectively. Using the Roseland approximation [6,9], $q_{rad}$ is expressed as $q_{rad}=-\frac{4\sigma^{*}}{3k^{*}}\left(\frac{\partial {Τ}^{4}}{\partial z}\right)$, here $\sigma^{*}$ (Stefan Boltzmann constant) as well as $k^{*}$ (absorption coefficient). We presume that the temperature of nanoliquid change inside stream is $T^{4}$ can be distributed in term of Taylor’s series. This occurs by expending $T^{4}$, nearly $T_{\infty }$ and the higher order term one of the form $T^{4}≅\;4\;\left(T_{\infty }^{3}T\right)-3\;T_{\infty }^{4},$ and $q_{rad}=-\frac{16\sigma^{*}}{3k^{*}}\frac{\partial T}{\partial z}.$ Therefore Equation (5) becomes

(9) $$\begin{array}{l} u\left(\frac{\partial \;T}{\partial \;x}\right)+v\left(\frac{\partial \;T}{\partial \;y}\right)+w\left(\frac{\partial \;T}{\partial \;z}\right)=\alpha_{nf}\left(\frac{\partial^{2}\;T}{\partial \;z^{2}}\right)+\frac{1}{{\left(\rho \;C_{p}\right)}_{nf}}[\left(\frac{16\sigma^{*}}{3k^{*}}\frac{\partial^{2}T}{\partial z^{2}}\right)+Q(T-T_{0})\\ +\frac{\sigma_{nf}\;B_{0}^{2}\;}{\left(m_{e}^{2}+n_{e}^{2}\right)}\left(u^{2}+v^{2}\right)+\frac{\mu }{{\left(\rho c_{p}\right)}_{nf}}{\left(\frac{\partial u}{\partial z}\right)}^{2}]. \end{array}$$

The suitable similarity transformations used in the present problem to change the system of PDEs Equations (2)–(5) into a set of ODEs are mostly used in various research articles [19,23]:

(10) $$\begin{array}{l} u=\left(s\;x\right)\;f^{\prime }(\eta ),\;v=\left(s\;x\right)\;g(\eta ),\;w=-\left(\sqrt{s\;\upsilon_{f}}\right)f(\eta ),\\ T-T_{\infty }=\left(T_{f}-T_{\infty }\right)\Theta (\eta ),\;\eta =\sqrt{\frac{s}{\upsilon_{f}}}. \end{array}$$

After utilizing the similarity parameters and relationship for nanoliquid, the Continuity equation is automatically verified while the resulting ODEs are:

(11) $$\begin{array}{l} \frac{f^{\prime \prime \prime }\left(\eta \right)}{{\left(1-\chi \right)}^{\frac{5}{2}}\left\{1-\chi +\frac{\rho_{CNT}}{\rho_{f}}\chi \right\}}-{\left[f^{\prime \prime }\left(\eta \right)\right]}^{2}+f\left(\eta \right)f^{\prime \prime }\left(\eta \right)-\lambda^{-1}\;\left[f^{\prime }\left(\eta \right)\right]-2\;\varepsilon g\left(\eta \right)\\ -\frac{{\left(1-\chi \right)}^{\frac{5}{2}}M}{\left(m_{e}^{2}+n_{e}^{2}\right)}\left(m_{e}f\left(\eta \right)-n_{e}g\left(\eta \right)\right)=0, \end{array}$$

(12) $$\begin{array}{l} \frac{g^{\prime \prime }\left(\eta \right)}{{\left(1-\chi \right)}^{\frac{5}{2}}\left\{1-\chi +\frac{\rho_{CNT}}{\rho_{f}}\chi \right\}}+f^{\prime }\left(\eta \right)\;g\left(\eta \right)+g^{\prime }\left(\eta \right)\;f\left(\eta \right)-\lambda^{-1}\;\left[g\left(\eta \right)\right]-2\;\varepsilon f^{\prime }\left(\eta \right)\\ +\frac{{\left(1-\chi \right)}^{\frac{5}{2}}M}{\left(m_{e}^{2}+n_{e}^{2}\right)}\left(n_{e}f^{\prime }\left(\eta \right)-m_{e}g\left(\eta \right)\right)=0, \end{array}$$

(13) $$\left(\frac{k_{nf}}{k_{f}}-\frac{4Rd}{3}\right)\Theta^{\prime \prime }\left(\eta \right)+Pr\left[\begin{array}{l} \left(1-\chi +\frac{{\left(\rho C_{p}\right)}_{CNT}}{{\left(\rho C_{p}\right)}_{f}}\chi \right)\left(f\left(\eta \right)\Theta^{\prime }\left(\eta \right)+H\Theta \left(\eta \right)\right)+\frac{Ec\;M}{\left(m_{e}^{2}+n_{e}^{2}\right)}\\ \left({f^{\prime }}^{2}\left(\eta \right)-g^{2}\left(\eta \right)\right)+Ec\;{f^{\prime \prime }}^{2}\left(\eta \right) \end{array}\right]=0.$$

(14) $$\begin{array}{l} f^{\prime }\left(0\right)=\;1,\;f\left(0\right)=0=g\left(0\right)\;,\;\Theta^{\prime }\left(0\right)=\frac{k_{f}}{k_{nf}}Bi\left(1-\Theta \left(0\right)\right),\\ f^{\prime }\;\left(\infty \right)\;\to \;0,\;g\;\left(\infty \right)\;\to \;0,\;\Theta \;\left(\infty \right)\;\to \;0. \end{array}$$

Here, ε, M, Rd, H   and   λ  while Pr,   Ec,   Bi, implies the change form of rotation, magnetic, radiation, heat generation/absorption and porosity parameters, while the Prandtl, Eckert and Biot numbers are respectively defined as

(15) $$\begin{array}{l} \varepsilon =\frac{\varpi }{s},M=\frac{\sigma_{f}B_{0}{}^{2}g}{s},Rd=\frac{16\;\sigma^{*}{\;T}_{\infty }^{3}}{3k^{*}k_{f}},Pr=\frac{{\left(\rho \;C_{p}\right)}_{f}}{k_{f}},\lambda \;=\frac{\rho_{f}K^{⊗}s}{\mu_{f}}\;,\;Ec=\frac{s^{2}\;x^{2}}{C_{p}\left(T_{f}-T_{\infty }\right)},\\ Bi=\frac{h_{f}}{k_{f}}\sqrt{\frac{\upsilon_{f}}{s}},\;\;H=\frac{Q}{{\left(\rho C_{p}\right)}_{f}}. \end{array}$$

The essential quantities of phenomenal and engineering significance are the coefficient of Skin friction $\left(C_{f}\right)$ in x,y−directions and the local Nusselt number (Nu) are explained as

(16) $$C_{fx}=\frac{1}{{\left(1-\varphi \right)}^{2.5}}\left(f^{\prime \prime }\left(0\right)\right),\;C_{fy}=\frac{1}{{\left(1-\varphi \right)}^{2.5}}\left(g^{\prime }\left(0\right)\right),\;Nu=-\frac{k_{nf}}{k_{f}}\left(\Theta^{\prime }\left(0\right)+Rd\right).$$

## 3. Homotopy Analysis Solution

For the HAM scheme we need the initial estimate $f_{0}(\eta ),\;g_{0}(\eta ),\;\Theta_{0}(\eta )$ and then the auxiliary linear operators, taking $\left(L_{f},\;L_{g},\;L_{\Theta }\right)$ for velocities and energy equations in the form

(17) $$f_{0}(\eta )=1-\frac{1}{e^{x}},\;g_{0}(\eta )=\frac{1}{e^{x}},\;\Theta_{0}(\eta )=\left(\frac{k_{f}Bi}{k_{f}Bi-k_{nf}}\right)\frac{1}{e^{x}}.$$

where L are

(18) $$L_{\;f}\;(f)=f_{\;\eta \;\eta \;\eta },\;\;L_{g}(g)=g_{\eta \;\eta }\;,\;L_{\;\Theta }(\Theta )=\Theta_{\eta \;\eta }.$$

The aforesaid stated operators are exposed as

(19) $$L_{f}(k_{1}+\left(k_{2}\right)\eta +\left(k_{3}\right)\eta^{2})=0,L_{g}(k_{4}+\left(k_{5}\right)\eta )=0,L_{\Theta }(k_{6}+\left(k_{7}\right)\eta )=0.$$

In which $k_{m}\;\left(m=1-7\right)$ denoted the approximate parameters. The 0-th and j-th order component of system are planned under: 

### 3.1. 0-th Order Set of System

Since r∈  [0, 1] as an inserting element using the concept of auxiliary factors $\hbar_{f},\hbar_{g},\hbar_{\Theta }$. Then problem deform for zero order as

(20) $$(1-r)\left[L_{f}\left(f(\eta ,\;r)-f_{0}(\eta )\right)\right]=r\;\hbar_{f}N_{f}\;\left[f\;(\eta ,r),\;g\;(\eta ,r)\right],$$

(21) $$(1-r)\left[L_{g}\;\left(g\;(\eta ,\;r)-g_{0}\;(\eta )\right)\right]=r\;\hbar_{g}N_{g}\;\left[f\;(\eta ,\;r),\;g\;(\eta ,r)\right],$$

(22) $$(1-r)\;\left[L_{\Theta }\left(\Theta (\eta ,r)-\Theta_{0}(\eta )\right)\right]=r\hbar_{\Theta }N_{\Theta }\left[f(\eta ,r),\;g(\eta ,r),\;\Theta (\eta ,r)\right].$$

Also

(23) $$\begin{array}{l} f\;(0,\;r)=0=g\;(0,\;r),\;f^{\prime }\;(0,\;r)=0,\;\Theta \;(0,\;r)=0,\\ f\;(1,\;r)=0=g\;(1,\;r),\;f^{\prime }\;(1,\;r)=0,\;\Theta \;(1,\;r)=0. \end{array}$$

We obtained the resulting non-linear operators in the following form:

(24) $$\begin{array}{l} N_{f}\left(f(\eta ;r),\;g(\eta ;\;r)\right)=\frac{\left[f_{\eta \eta \eta }\;(\eta \;;\;r)\right]}{{\left(1-\chi \right)}^{\frac{5}{2}}\left\{1-\chi +\frac{\rho_{CNT}}{\rho_{f}}\chi \right\}}+[\frac{1}{{\left(f_{\eta \eta }\;(\eta ;\;r)\right)}^{-2}}+f_{\eta \eta }(\eta ;\;r)\left(f(\eta ;\;r)\right)\;\\ +\frac{1}{\lambda }\left[f^{\prime }(\eta ;\;r)\right]+2\;\varepsilon \;g(\eta ;\;r)-\frac{{\left(1-\chi \right)}^{\frac{5}{2}}M}{\left(m_{e}^{2}+n_{e}^{2}\right)}\left(m_{e}\;f(\eta ;\;r)-n_{e}\;g(\eta ;r)\right)], \end{array}$$

(25) $$\begin{array}{l} N_{g}\left(g(\eta ;r),\;\;f(\eta ;r)\right)=\frac{\left[g_{\eta \eta }\;(\eta \;;\;r)\right]}{{\left(1-\chi \right)}^{\frac{5}{2}}\left\{1-\chi +\frac{\rho_{CNT}}{\rho_{f}}\chi \right\}}-[f_{\eta }\;(\eta \;;\;r)\;g(\eta ;\;r)+f(\eta ;\;r)\;g_{\eta }(\eta ;\;r)\\ -\frac{1}{\lambda }\;\left[g(\;\eta ;\;r)\right]-2\;\varepsilon \left[f_{\eta }\;(\eta ;\;r)\right]+\frac{{\left(1-\chi \right)}^{\frac{5}{2}}M}{\left(m_{e}^{2}+n_{e}^{2}\right)}\left(n_{e}\;f^{\prime }(\eta ;\;r)-m_{e}\;g(\eta ;\;r)\right), \end{array}$$

(26) $$\begin{array}{l} N_{\Theta }\;\left(\Theta \;(\eta ;\;r),\;f(\eta ;\;r),\;\Phi (\eta ;\;r)\right)=\left(\frac{k_{nf}}{k_{f}}-\frac{4Rd}{3}\right)\;\Theta_{\eta \eta }(\eta ;\;r)+Pr[\left(1-\chi +\frac{{\left(\rho C_{p}\right)}_{CNT}}{{\left(\rho C_{p}\right)}_{f}}\chi \right)\\ \left(f(\eta ;\;r)\;\Theta_{\eta }\;(\eta ;\;r)+H\;\Theta \;(\eta ;\;r)\right)+\frac{Ec\;M}{\left(m_{e}^{2}+n_{e}^{2}\right)}\left(f_{\eta }{}^{2}(\eta ;\;r)-g^{2}(\eta ;\;r)\right)+Ecf_{\eta \eta }^{2}(\eta ;\;r)]. \end{array}$$

From Taylor’s series, express  f(η;r), g(η;r), Θ(η;r)  in terms of r:

(27) $$f(\eta ;r)=f_{0}(\eta )+\sum_{j=1}^{\infty }f_{j}(\eta ),g(\eta ;r)=g_{0}(\eta )+\sum_{j=1}^{\infty }g_{j}(\eta ),\;\Theta (\eta ;r)=\Theta_{0}(\eta )\;+\sum_{j=1}^{\infty }\Theta_{j}(\eta ).$$

where 

(28) $$\;f_{j}(\eta )={\frac{1}{j!}\left(f_{\eta }(\eta ;r)\right)|}_{r=0},\;g_{j}(\eta )={\frac{1}{j!}\left(g_{\eta }(\eta ;r)\right)|}_{r=0},\;\;\Theta_{i}(\eta )={\frac{1}{j!}\left(\Theta_{\eta }(\eta ;r)\right)|}_{r=0}.$$

### 3.2. j^th^-Order Deformation System

(29) $$\begin{array}{l} L_{f}\left(f_{j}\;(\eta )-\zeta_{j}f_{j-1}\;(\eta )\right)=\hbar_{f}\left(R_{j}^{f}(\eta )\right),\;\;L_{g}\left(g_{j}\;(\eta )-\zeta_{j}g_{j-1}\;(\eta )\right)=\hbar_{g}\left(R_{j}^{g}(\eta )\right),\\ L_{\Theta }\left(\Theta_{j}\;(\eta )-\zeta_{j}\Theta_{j-1}\;(\eta )\right)=\hbar_{\Theta }\left(R_{j}^{\Theta }(\eta )\right). \end{array}$$

and

(30) $$\begin{array}{l} {f^{\prime }}_{j}=f_{j}=g_{j}=\Theta_{j}=0,\;at\;\eta =0,\\ {f^{\prime }}_{j}=f_{j}=g_{j}=\Theta_{j}=0\;at\;\eta =1. \end{array}$$

(31) $$\begin{array}{l} R_{j}^{f}(\eta )=\frac{f_{j-1}^{\prime \prime \prime }}{{\left(1-\chi \right)}^{\frac{5}{2}}\left\{1-\chi +\frac{\rho_{CNT}}{\rho_{f}}\chi \right\}}+[{\left(\sum_{k=1}^{j-1}\;\left(f_{j-1}^{\prime \prime }\right)\right)}^{2}+\left(\sum_{k=1}^{j-1}\;\left(f_{j-1-k}\right)\;f_{k}^{\prime \prime }\right)+\frac{1}{\lambda }\;\left(\sum_{k=1}^{j-1}\;\left(f_{j-1}^{\prime }\right)\right)\\ +2\varepsilon g_{j-1}-\frac{{\left(1-\chi \right)}^{\frac{5}{2}}M}{\left(m_{e}^{2}+n_{e}^{2}\right)}\left(m_{e}f_{j-1}-n_{e}g_{j-1}\right)], \end{array}$$

(32) $$\begin{array}{l} R_{j}^{f}(\eta )=\frac{g_{j-1}^{\prime \prime }}{{\left(1-\chi \right)}^{\frac{5}{2}}\left\{1-\chi +\frac{\rho_{CNT}}{\rho_{f}}\chi \right\}}+[\left(\sum_{k=1}^{j-1}\;\left(\;f_{j-1}^{\prime }.g_{k}\right)\right)+\left(\sum_{k=1}^{j-1}\;f_{j-1-k}.\;g_{k}^{\prime }\right)-\frac{1}{\lambda }\left[g_{j-1}\right]\\ -2\;\varepsilon \;\left(\sum_{k=1}^{j-1}\;f_{j-1}^{\prime }\right)+\frac{{\left(1-\chi \right)}^{\frac{5}{2}}M}{\left(m_{e}^{2}+n_{e}^{2}\right)}\left(n_{e}\;\sum_{k=1}^{j-1}\;f_{j-1}^{\prime }-m_{e}\;g_{j-1})\right)], \end{array}$$

(33) $$\begin{array}{l} R_{j}^{f}(\eta )=\left(\frac{k_{nf}}{k_{f}}-\frac{4Rd}{3}\right)\Theta_{j-1}^{\prime \prime }+Pr[\left(1-\chi +\frac{{\left(\rho C_{p}\right)}_{CNT}}{{\left(\rho C_{p}\right)}_{f}}\chi \right)\left(f_{j}\sum_{k=1}^{i-1}\Theta_{j-1}^{\prime }+H\Theta_{k}\right)\\ +\frac{Ec\;M}{\left(m_{e}^{2}+n_{e}^{2}\right)}\left({\sum_{k=1}^{j-1}\left(f_{j-1-k}^{\prime }\right)}^{2}-g^{2}{}_{k}\right)+Ec\;{\sum_{k=1}^{j-1}\left(f_{j-1}^{\prime \prime }\right)}^{2}\left(\eta \right)]. \end{array}$$

where

(34) $$\zeta_{j}=\left\{ \begin{array}{l} 1,\;\mathrm{if}\;\;r>1\\ 0,\;\mathrm{if}\;\;r\leq 1\; \end{array}\right..$$

### 3.3. The Convergence of Homotopy Result

In the construction of the series solution, the auxiliary variable $\hbar_{f},\hbar_{g},\hbar_{\Theta }$ is very important. These variables play a major part in regulating and governing the convergence region of the solution. These figures displaying the applicable values of $\hbar_{f},\hbar_{g},\hbar_{\Theta }$ after 18-th order of approximation are in the ranges of as $-1.5\leq \hbar_{f}\leq -0.2,-1.4\leq \hbar_{g}\leq -0.3,-1.5\leq \hbar_{\Theta }\leq -0.3$ for SWCNT, and $-1.5\;\leq \;\hbar_{f}\leq -\;0.3,\;-1.3\;\leq \;\hbar_{g}\leq -0.4,\;-1.5\;\leq \;\hbar_{\Theta }\leq -0.2$ for MWCNT.

## 4. Results and Discussion 

The current section presents the impact of some emerging model variables on velocities $\left(f^{\prime }\left(\eta \right),\;g\left(\eta \right)\right)$ and the thermal field Θ(η) for both cases of nanoparticles, single and multi-walled CNTs nanoliquids. These emerging specific model variables are the rotating parameter (ε), magnetic parameter (M), nanoparticle volume fraction (χ), Prandtl number (Pr), Eckert (Ec) numbers, radiation factor (Rd), local porosity parameter (λ), Biot number (Bi), heat generation/absorption factor (H), Hall factor $\left(n_{e}\right)$, and ion-slip factor $\left(n_{i}\right).$ The graphical view and coordinates axis of the problem are presented in Figure 1.

**Figure 2:**
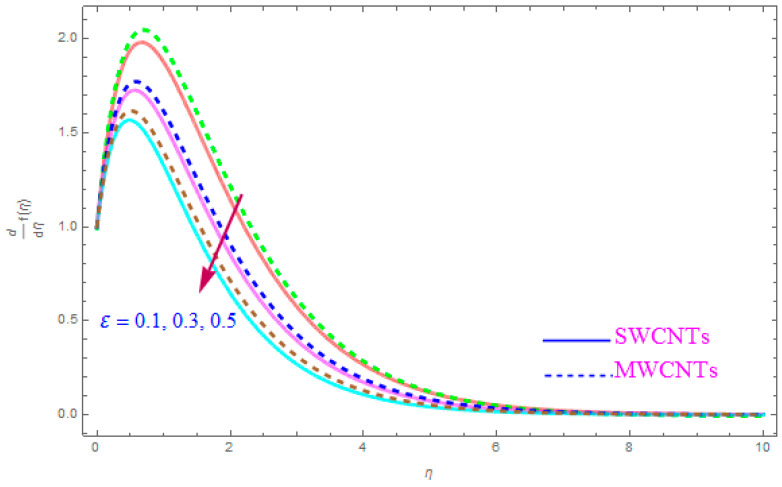
Plots the influence of $f^{\prime }\left(\eta \right)$ for rotation parameter (ε).

**Figure 3:**
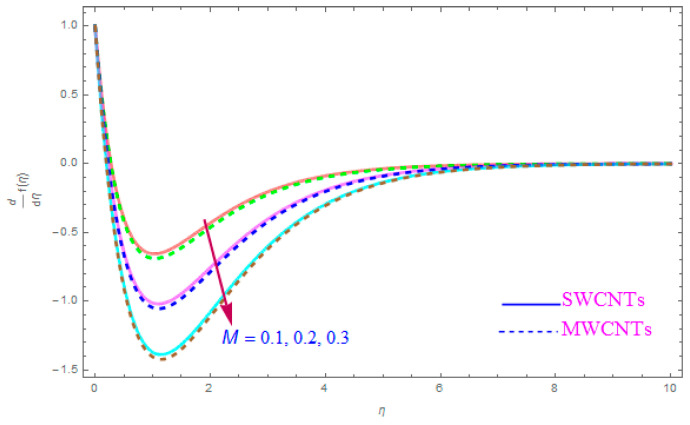
Plots the influence of $f^{\prime }\left(\eta \right)$ for magnetic parameter (M).

**Figure 4:**
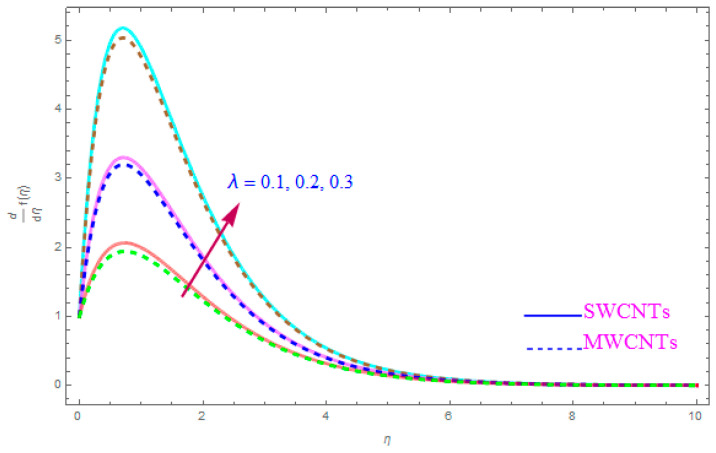
Plots the influence of $f^{\prime }\left(\eta \right)$ for porosity parameter (λ).

**Figure 5:**
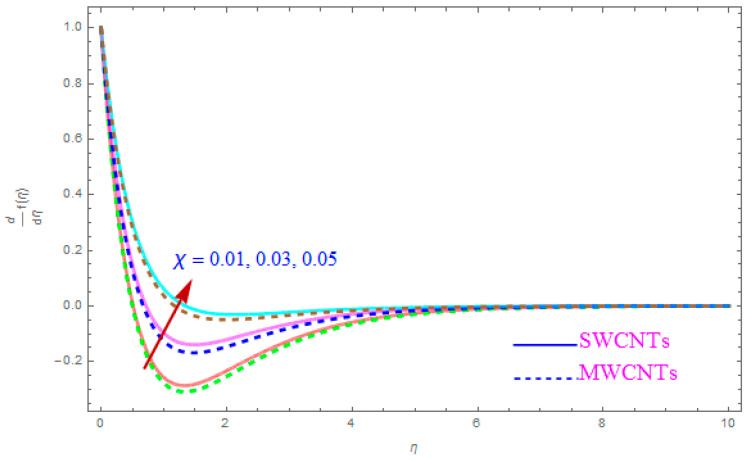
Plots the influence of $f^{\prime }\left(\eta \right)$ for nanoparticle volume fraction (χ).

**Figure 6:**
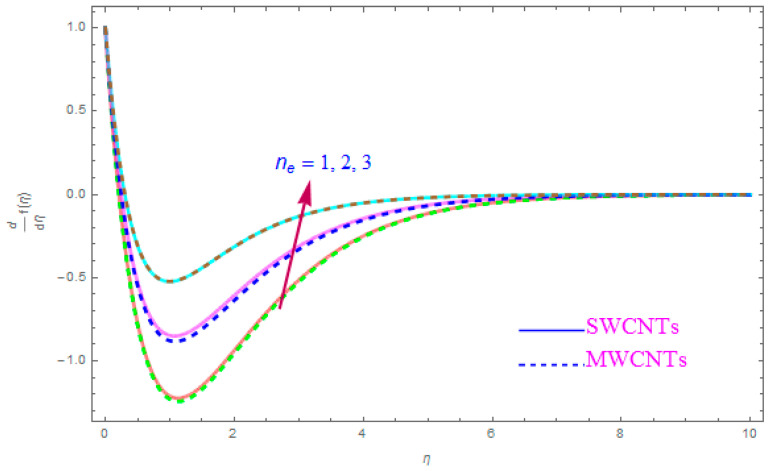
Plots the influence of $f^{\prime }\left(\eta \right)$ for Hall parameter $\left(n_{e}\right)$.

**Figure 7:**
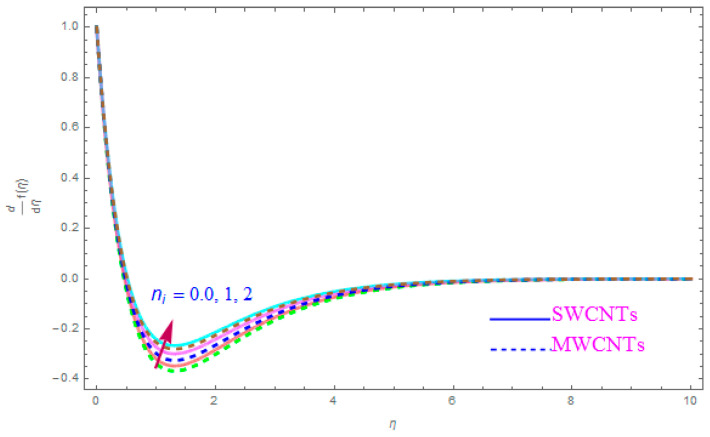
Plots the impact of $f^{\prime }\left(\eta \right)$ for ion-slip parameter $\left(n_{i}\right)$.

The influence of such model parameters $\varepsilon ,M,\;\lambda ,\;\chi ,\;n_{e},\;n_{i}$ on x-component of velocity $f^{\prime }\left(\eta \right)$ has been sketched in Figure 2, Figure 3, Figure 4, Figure 5, Figure 6 and Figure 7 and deliberated its consequence. The influence of the rotational parameter (ε) on $f^{\prime }\left(\eta \right)$ (x-component of velocity) is presented in Figure 2. From the assessment of the sketch, we perceived that by accelerating the value of (ε), the x-component of velocity field $f^{\prime }\left(\eta \right)$ shows the declining tendency for both nanoparticles SWCNT and MWCNT nanoliquids. Actually, the large magnitude of the rotating factor (ε) reduces the nanofluid velocity and as a consequence the fluid is moving as well as rotating, so the rotated opposing force is therefore produced by the flow. In the situation of high scale of the (ε) variable, the rate of fluid rotation becomes dominant as equated to the sheet stretching frequency. The high rate of rotation produces resistance to the liquid motion, so $f^{\prime }\left(\eta \right)$ shows the decreasing behavior. The inspection of Figure 2 also shows that the velocity of SWCNT is obviously lower in magnitude then that of the MWCNTs due to the high density of SWCNTs as MWCNTs. Therefore the $f^{\prime }\left(\eta \right)$ of SWCNT reduces more readily than that of MWCNTs. Figure 3 shows the continuous impact of magnetic parameter (M) on the x-component of velocity distribution $f^{\prime }\left(\eta \right)$ for both cases of SWCNTs and MWCNTs nanoparticles. The growing power of the magnetic factor (M) declines the velocity of the nanoliquid. In fact, when we enhance the strength of (M), as a result a resistive form of force (drag force) known as the Lorentz force contributes dynamically and this resistive force offers opposition to the flow of liquid elements, which carries debility in the horizontal velocity profile $f^{\prime }\left(\eta \right)$. Clearly, from the assessment of graphs displays that the velocity of SWCNT is rapidly reduce in magnitude then that of MWCNTs. Figure 4 exhibits the consequence of the local permeability (porosity) parameter (λ) on the velocity field $f^{\prime }\left(\eta \right)$ for SWCNT as well as MWCNT nanoliquids. It is witnessed that raising the value of porosity factor leads to improvement of the velocity distribution. The basic fact is that the spongy surface influence on the boundary film’s progress is very significant due to strengthening the viscosity of the thermic boundary layer. Therefore, it is observed that enlarging the permeability of medium leads to a quicker movement of nanoliquid on it. Physically, by extending the dumps of the porous medium, the resistance of the porous medium may be omitted. The analysis of the graphs shows that the velocity of SWCNT is noticeably faster then that of the MWCNTs nanoparticles. The impact of the (χ) nanoparticle volume fraction parameter on $f^{\prime }\left(\eta \right)$ is exposed in Figure 5. From the sketch it is perceived that $f^{\prime }\left(\eta \right)$ of nanofluid and allied thickness of the stream is accelerated for both nanoparticles. Furthermore, from the graph it is observed that the velocity of the liquid small particle is leading in case of SWCNTs, as compared to MWCNTs. Figure 6 clearly shows the impact of the Hall parameter $\left(n_{e}\right)$ on $f^{\prime }\left(\eta \right)$ It is witnessed from the figure that the velocity field rises for large magnitude of the Hall parameter $\left(n_{e}\right)$ for (S and MWCNT) nanoparticles. Consequently, the presence of Hall variable $\left(n_{e}\right)$ reduces the conflicting force provided by the applied magnetic field. Hence $f^{\prime }\left(\eta \right)$ increases as the value of $\left(n_{e}\right)$ accelerates, but this increment in $f^{\prime }\left(\eta \right)$ is very small. Similarly Figure 7 is drawn to interpret the significance of the ion-slip variable $\left(n_{i}\right)$ on the horizontal velocity $f^{\prime }\left(\eta \right)$. It is shown that the $f^{\prime }\left(\eta \right)$ (velocity filed) rises for bulky magnitude of ion-slip parameter $\left(n_{i}\right)$ for both SWCNT and MWCNT nanoliquids. Generally, with the addition of Hall parameter along with ion-slip factor, the speed of liquid stream increases, which enhances the viscosity of the boundary film. Since the effective conductivity improves as the value of the ion-slip parameter $\left(n_{i}\right)$ increases, therefore the damping force over the velocity of fluid is reduced for both kerosene oil-based nanoparticles. 

**Figure 8:**
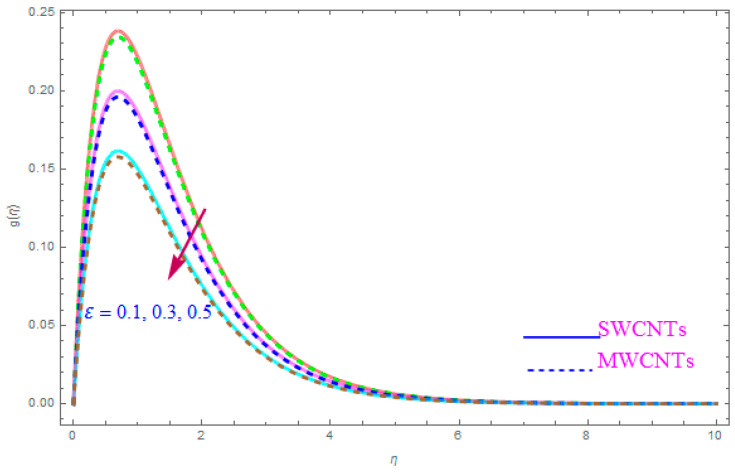
Plots the impact of g(η) for rotation parameter (ε).

**Figure 9:**
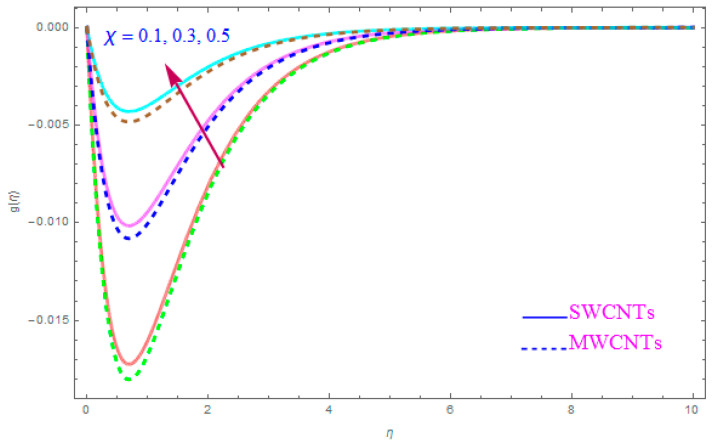
Plots the influence of g(η) for nanoparticle volume fraction (χ).

**Figure 10:**
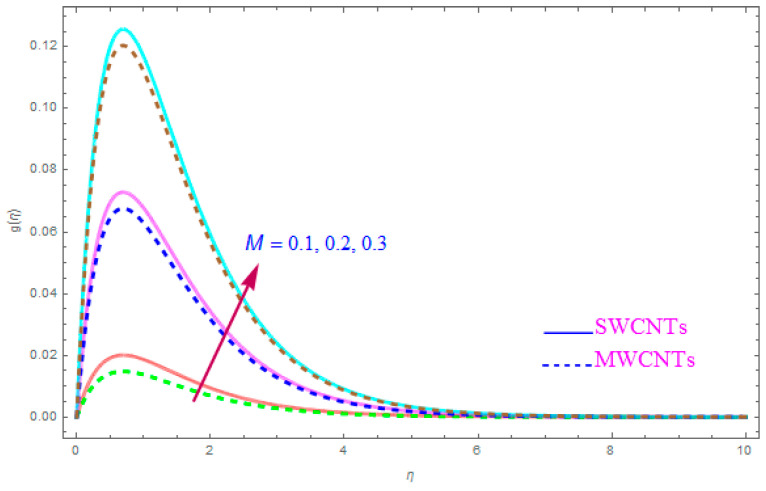
Plots the influence of g(η) for magnetic parameter (M).

**Figure 11:**
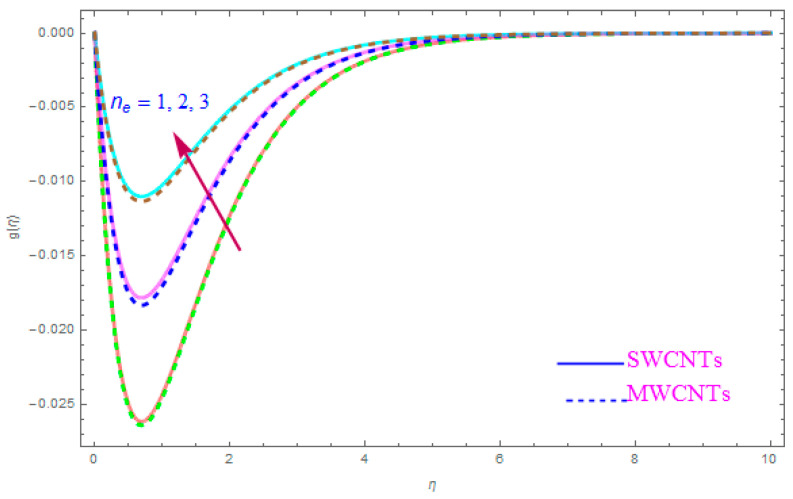
Plots the influence of g(η) for Hall parameter $\left(n_{e}\right)$.

The impact of model parameters like $\varepsilon ,M,\;ƛ,\;\chi ,\;n_{e},\;n_{i}$ on g(η) has been provided in Figure 8, Figure 9, Figure 10 and Figure 11, which show its significance for both types of kerosene oil based nanoparticles. The importance of rotational parameter (ε) on the g(η) y-component velocity field is visually presented in Figure 8. This figure displays that, for both SWCNT and MWCNT nanoliquids, the fluid velocity declines while increasing the magnitude of the rotation parameter (ε). The present sketch also indicates that the reduction in the y component of velocity is more than in the x-component of velocity. In fact, this shows that the delaying force along the y direction is more than in the x direction. The behavior of χ (nanoparticles volume fractions factor) on y-component of velocity distribution g(η) is portrayed in Figure 9. From the figure, it is examined that the velocity improves through the higher approximation of χ (nanoparticles volume fractions). Gradually the g(η) velocity and viscosity of the boundary film are enhanced for both cases of CNTs nanoliquid. Figure 10 shows the influence of magnetic variable (M) on g(η). It is observed that an increment in strength of (M) leads to a lower g(η) for both types of (S and MWCNT) nanoliquids. The velocity of liquid in the y-direction flow rises in the neighborhood of the sheet, but this leads to extreme declines in boundary film section from the sheet to the large magnitude of (M) magnetic parameter. In fact, this is because the enforced applied $B_{0}$ (magnetic field) yields a resistive power in the shape of Lorentz’s force, which is produced with the wave of magnetically charge elements, which reduces the value of g(η). Figure 11 is impersonated to recognize the consequence of the Hall parameter $\left(n_{e}\right)$ on g(η). From the figure, it can be seen that the g(η) improves with a high magnitude of the Hall parameter $\left(n_{e}\right)$ for both CNTs (S and MWCNTs). Physically, the existence of $\left(n_{e}\right)$ decays the contrasting force performed by means of the applied magnetic field. 

**Figure 12:**
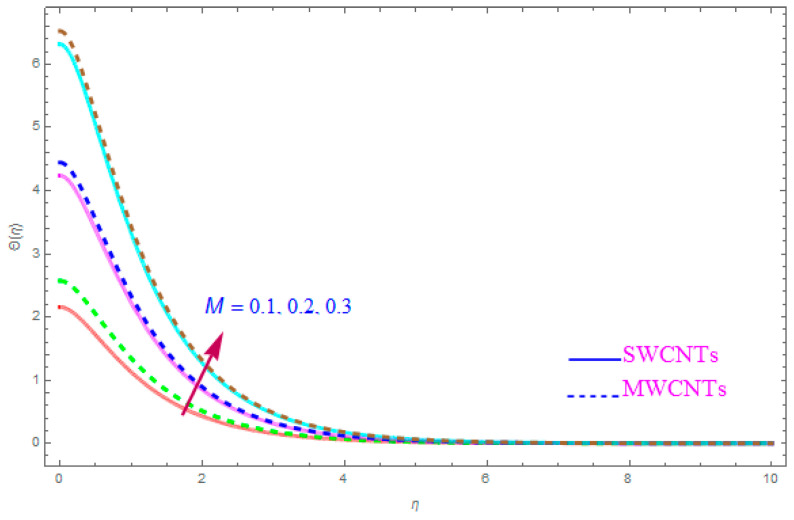
Plots the influence of thermal field Θ(η) for magnetic parameter (M).

**Figure 13:**
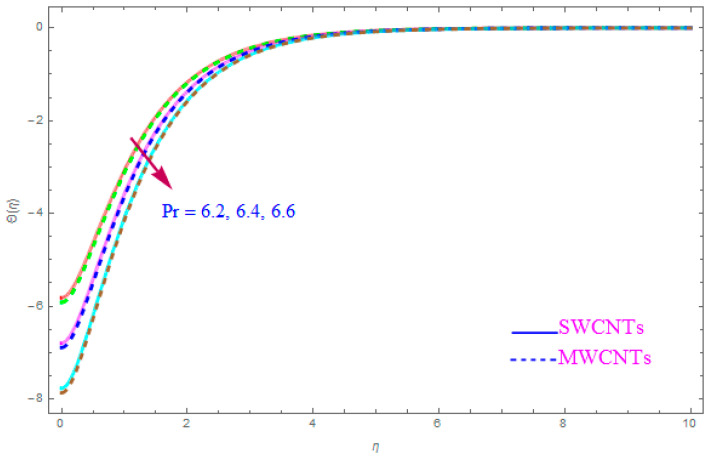
Plots the influence of thermal field Θ(η) for Prandtl number (Pr).

**Figure 14:**
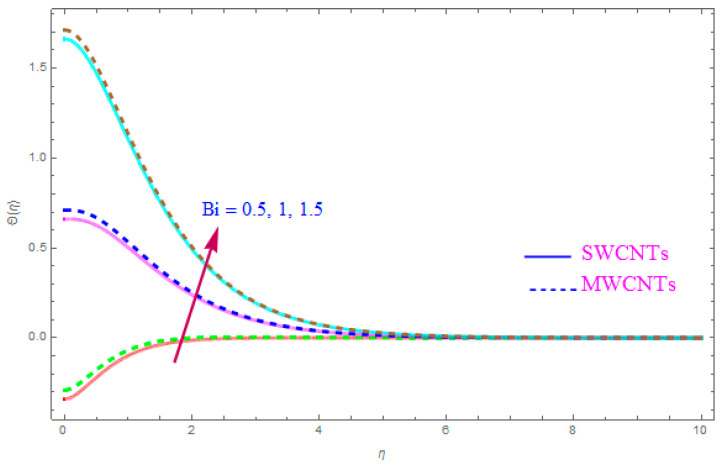
Plots the influence of thermal field Θ(η) for Biot number (Bi).

**Figure 15:**
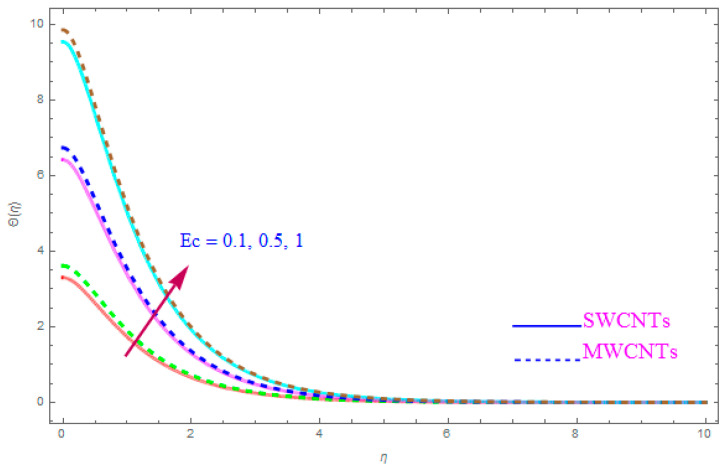
Plots the influence of thermal field Θ(η) for Eckert number (Ec).

**Figure 16:**
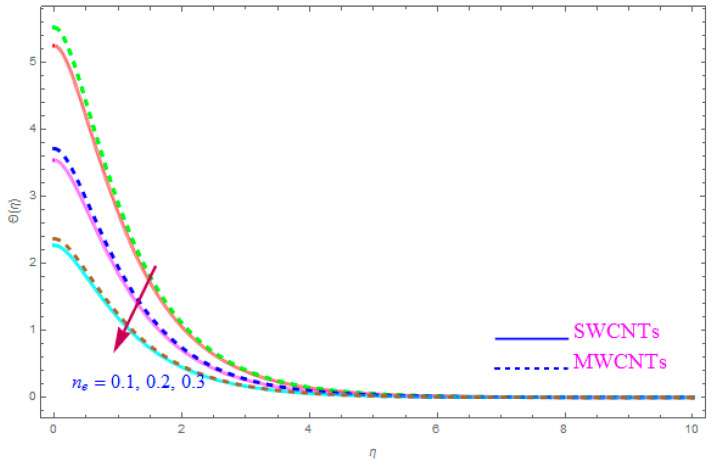
Plots the influence of thermal field Θ(η) for Hall parameter $\left(n_{e}\right).$

**Figure 17:**
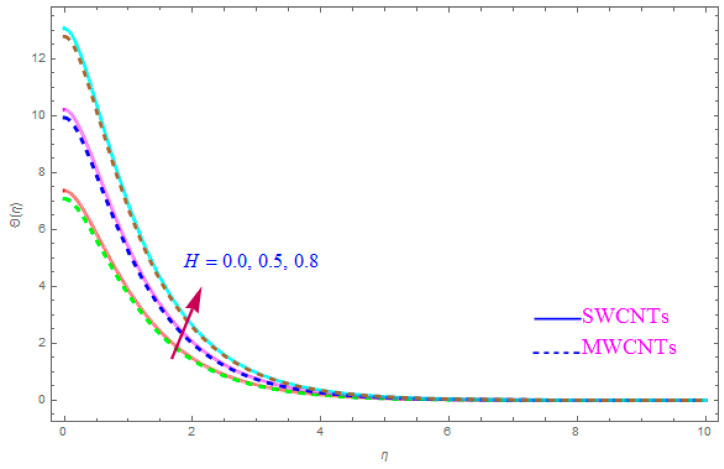
Plots the influence of thermal field Θ(η) for heat generation (H>0) parameter.

**Figure 18:**
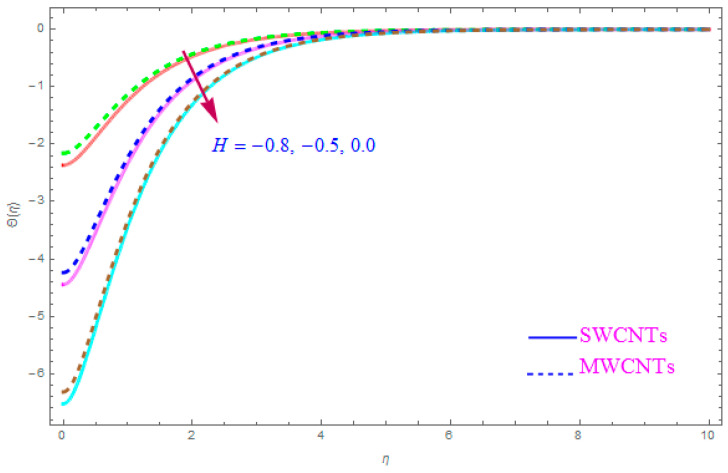
Plots the influence of thermal field Θ(η) for heat absorption (H<0) parameter.

Figure 12 is shown to understand the outcome of magnetic (M) parameter on the thermic field Θ(η). It is noted that the thermal filed Θ(η) enhance for large value of magnetic factor (M) for both SWCNT-kerosene and MWCNT-kerosene nanofluids. Actually, we notice that the temperature of boundary films becomes dense (thick) as the magnitude of (M) increases. The impact of a crosswise magnetic field provides an increase to an opposing effort identified as the “Lorentz force”. Such a strong opposing effort has the ability to make the flow of the liquid sluggish and increase its thermal boundary films, therefore increasing the Θ(η) of the flow. Figure 13 highlights the consequence of Prandtl number (Pr) on the thermal field Θ(η). A decreasing conduct of thermal field Θ(η) is detected for enhancement in the Prandtl number (Pr) of both (S and MWCNT) nanoliquids. It is noted that an accelerating rate of Prandtl number (Pr) yields a lesser temperature boundary film viscosity (thickness). Physically, liquids having a higher magnitude of Prandtl number (Pr) have lower thermal diffusivity, and therefore, the temperature of liquid declines. Also, a stronger thermal film is observed for both sorts of CNTs. Figure 14 scrutinizes the way that the Biot number (Bi) affected the thermal field Θ(η). From the diagram, we can observe that the thermal filed and the thermic boundary film thickness were both enhanced for a higher Biot number (Bi). Therefore, a high rate of the Biot number (Bi) generates cooling in the nanofluid. The characteristics of the various values of the Eckert number (Ec) on Θ(η)(thermal profile) are explained in Figure 15. It is shown that Θ(η) profile enhances with a raise in (Ec) due to the involvement of the viscous dissipation for (Single and Multi) WCNTs. From the physical point of view, a high magnitude of (Ec) increases the temperature (thermal condition) of the sheet which is transfered to the flow of nanoliquid and consequently the temperature of internal liquid surges upwards. The consequence of Hall parameter $\left(n_{e}\right)$ on the thermal field Θ(η) is displayed in Figure 16. It is noted that the temperature profile decreases, but at the same time velocity profile increases due to the opposing force executed by the applied magnetic field. Figure 17 and Figure 18 sketched the influence of H>0 (Heat generation factor) and H<0 (Heat absorption factor) for both CNTs and kerosene nanofluid. From the plots, it is evident that (H>0) accelerates Θ(η) while (H<0) depreciates Θ(η). It is quite noticeable that the existence of (Single and Multi) WCNTs has enhanced the Θ(η) of the nanofluid while heat generation (H>0) exists in the structure, which leads to the Θ(η) increasing even further. But if the heat absorption (H<0) factor gains the inner heat energy from the sheet surface, then we obtain a reduction in Θ(η). These graphical results are qualitatively in strong agreement with the results of Bilal et al. [29]. 

### Table Discussion 

Table 1 delivers some physical characteristics (density, specific heat, and thermal conductivity) of base liquid (kerosene oil) and nanoparticles. Table 2 provides the excellent converging of the homotopy outcomes for the 18^th^ order of estimations. Furthermore, Table 3 has been planned to show the effects of some physical model variables like $\chi ,\;\lambda ,\;M,\;\varepsilon ,\;\;m_{e},\;n_{e}$ on $-f^{\prime \prime }(0)$ (surface drag force) for both nanoliquids. It has been observed from the mathematical computation that $-f^{\prime \prime }(0)$ shows a declining behavior due to the increasing value of ε (rotating parameter) which presents a strong opposing type force that is essential for opposing the liquid flow through the extending surface. Meanwhile, $-f^{\prime \prime }(0)$ improves with increasing values of other parameters $\chi ,\;\lambda ,\;M,\;\;m_{e},\;n_{e}$. By increasing the value of χ (nanoparticle volume fraction) the allied thickness of the stream is accelerated, so the velocity of tiny particles also accelerated. The $-f^{\prime \prime }(0)$ (surface drag force) is higher for MWCNT when it is equated to SWCNT. Similarly, Table 4 has been planned to determine the impact of some model variables like Rd, Pr, Ec, λ on Nu (heat transport rate) for both SWCNT and MWCNT nanoliquids. It has been observed from numerical computation that the rate of heat transport shows a decreasing tendency due to the increasing value of Rd, which presents a strong opposing type force that is essential for opposing the liquid flow through the stretching sheet.

## 5. Conclusions 

In this work, the influence of two types of nanometer sized particles, single and multi WCNTs on three dimensional rotating flows through porous stretching sheet is studied in terms of the combined effect of Hall current, ion-slip, and the thermal radiation effect. The main findings of present flow problem are summarized as the following:

Both velocities fields $(f^{\prime }\left(\eta \right)$ and g(η)) depict increasing behavior for large value of the CNTs nanoparticle. Similarly, the greater density of nanoparticles delivers a higher rate of heat transport. 

The velocity field $(f^{\prime }\left(\eta \right)$ of (Single and Multi) WCNTs exhibited an increasing trend for a high amount of (χ), the Hall parameter $\left(n_{e}\right)$, and ion-slip parameter $\left(n_{i}\right)$. However, the opposite behavior is noted for the high value of rotation and the (M) magnetic parameter.

The g(η) velocity field displays an enhanced trend for large magnitudes of M,χ, $n_{e}$, $n_{i}$; however, the velocity drops with the rising value of the rotation parameter.

In both CNTs temperature filed of nanofluid and boundary layer is decreasing function of Hall parameter $\left(n_{e}\right)$ due to the opposite force performed by the applied magnetic field.

Intensification in Θ(η) (thermal field) has been noticed for leading to a higher value for the heat generation factor (H>0) while the opposite behavior has been observed for the rising value of the heat absorption factor (H<0).

M and Ec presents increasing behavior for temperature profile Θ(η) because a high magnitude of (Ec) increases the temperature of the sheet surface, which is transferred to the liquid flow and the temperature of the internal liquid therefore increases.

A large value of the Biot number (Bi) accelerates the thermal filed and makes the boundary film concentration thicker. 

Increments in Prandtl number (Pr) lead to a reduced Θ(η) for SWCNTs as well as MWCNTs.

Greater Ec increases the magnitude of Θ(η), so this process heats up nanofluid, which decreases the freezing effect for both CNTs.

The increasing value of χ,  M shows the augmented behavior of $-f^{\prime \prime }(0)$.

$-\frac{k_{nf}}{k_{f}}\Theta^{\prime }(0)$ is enhanced by using high magnitude of χ, Re,  λ,  Ec for nanoparticles.

These graphical results are qualitatively in excellent contract with results of Bilal et al. [29].

The role of MWCNTs is overriding that of SWCNTs.

## Figures and Tables

**Table 1:** Some basic thermo-physical characteristics of Kerosene oil and nanoparticles (SWCNT and MWCNT) [13,17,19].

Physical Properties	Base Liquid	Nanosize Particles	
	Kerosene Oil	SWCNT	MWCNT
$\rho \;\left({\mathrm{kgm}}^{-3}\right)$	783	2600	1600
$C_{p}\left[j{\left(\mathrm{kgK}\right)}^{-1}\right]$	2090	425	796
$k\left[w{\left(\mathrm{mK}\right)}^{-1}\right]$	0.145	6600	3000

**Table 2:** The convergence of the Homotopic outcomes for various order of estimations.

Order of Approximations	SWCNTs-Kerosene			MWCNTs-Kerosene		
	$f^{\prime \prime }\left(0\right)$	$g^{\prime }\left(0\right)$	$-\Theta^{\prime }\left(0\right)$	$f^{\prime \prime }\left(0\right)$	$g^{\prime }\left(0\right)$	$-\Theta^{\prime }\left(0\right)$
1	0.36550	0.19234	0.6847	0.29470	0.18531	0.50562
5	0.32521	0.19123	0.4667	0.25210	0.18323	0.39435
10	0.31063	0.18723	0.4023	0.24320	0.18103	0.36716
15	0.30217	0.18214	0.3931	0.23230	0.17214	0.33958
18	0.20132	0.18213	0.2299	0.12905	0.17202	0.30210

**Table 3:** Exhibits the mathematical values for the skin friction coefficient using various physical parameters.

ϕ	λ	M	Ω	$m_{e}$	$n_{e}$	${\left(1-\phi \right)}^{-2.5}f^{\prime \prime }(0)$		${\left(1-\phi \right)}^{-2.5}g^{\prime }(0)$	
						SWCNT	MWCNT	SWCNT	MWCNT
**0.1**	**0.2**	**0.5**	**0.2**	**0.1**	**0.1**	−1.93809	−1.90481	0.329385	0.30179
0.2						−2.34557	−2.23450	0.284000	0.221342
0.3						−2.86236	−2.74537	0.218497	0.20041
0.1	0.2					−1.93809	−1.90152	0.329385	0.31191
	0.3					−1.93809	−1.91145	0.329385	0.31192
	0.4					−1.93809	−1.91245	0.329385	0.31193
	0.2	0.5				−1.93809	−1.80341	0.329385	0.31193
		0.6				−1.97461	−1.92340	0.404418	0.39832
		0.7				−2.00896	−1.98921	0.476478	0.39241
		0.5	0.2			−1.93809	−1.90452	0.329385	0.30171
			0.3			−1.94134	−1.92345	0.285883	0.27343
			0.4			−1.94413	−1.93532	0.242621	0.20129
				0.1		−1.93809	−1.87231	0.329385	0.29817
				0.2		−1.90602	−1.85342	0.381362	0.31242
				0.3		−1.86140	−1.83425	0.395883	0.35241
				0.1	0.1	−1.93809	−1.83425	0.329385	0.30123
					0.2	−1.80716	−1.63420	0.261963	0.24231
					0.3	−1.76420	−1.53421	0.177045	0.21611

**Table 4:** Shows the mathematical values for Nu using various model factors.

Rd	Pr	Ec	λ	Nu	
				SWCNT	MWCNT
**0.3**	**6.4**	**0.2**	**0.2**	−0.0277452	−0.0240453
0.4				−0.0251504	−0.0221502
0.5				−0.0225163	−0.0155167
0.3	6.4			−0.0277452	−0.0257453
	6.6			−0.0138570	−0.0128575
	6.8			−0.00001796	−0.0001179
	6.4	0.2		−0.0277452	−0.0167453
		0.3		−0.0210052	−0.0134055
		0.4		−0.0697556	−0.0397554
		0.2	0.2	−0.0277452	−0.0247454
			0.3	−0.0268688	−0.0230681
			0.4	−0.0259934	−0.0220934

## Nomenclature

**List of Symbols**		**Greek Symbols**	
u, vw	Velocities components $\left({\mathrm{ms}}^{-1}\right).$	$\mu_{nf}$	Dynamic viscosity of nanofluid (mPa).
$B_{0}$	Magnetic field strength $\left({\mathrm{NmA}}^{-1}\right).$	$\mu_{f}$	Dynamic viscosity of base fluid (mPa).
f,g	Dimensional velocity profiles	$\rho_{nf}$	Nanofluid density $\left({\mathrm{Kgm}}^{-3}\right).$
T	Fluid temperature (K).	$\rho_{f}$	Base fluid density $\left({\mathrm{Kgm}}^{-3}\right).$
$T_{f}$	Temperature of base fluid (K).	η	Similarity variable.
$T_{\infty }$	Free surface temperature (K).	χ	Nanoparticle volume fraction
M	Magnetic parameter.	Θ	Dimensional heat profiles.
$h_{f}$	Heat transfer coefficient.	$\sigma_{nf}$	Electrical conductivity
s	Stretching parameter.	ω	Electron cyclotron
Pr	Prandtl number.	ϖ	Angular velocity
Bi	Biot number.	λ	Porosity parameter
Ec	Eckert number.	ℏ	Auxiliary constant
Νu	Nusselt number.	Subscripts	
$C_{f}$	Skin friction coefficient	nf	Nanofluid
$n_{e}$	Hall parameter	f	Base fluid
$n_{i}$	Ion-slip parameter	**Abbreviations**	
$u_{w}$	Stretching velocity	ODE	Ordinary differential equation
${\left(C_{p}\right)}_{f}$	Specific heat of base fluid (J/kgK).	HAM	Homotopy Asymptotic Method
		MHD	Magneto-hydrodynamics
$k_{nf}$	Thermal conductivity (${\mathrm{Wm}}^{-1}K^{-1}$).	SWCNT	Single wall carbon nanotube
H	Heat generation/absorption parameter	MWCNT	Multi wall carbon nanotube
$\sigma^{*}$	Stefan Boltzmann constant.	MRA	Magnetic resonance angiography
$k^{*}$	Absorption coefficient.	MRI	Magnetic resonance imaging
